# [(*Z*)-*N*,*O*-Disopropyl­thio­carbamato-κ*S*](tricyclo­hexyl­phosphine-κ*P*)gold(I)

**DOI:** 10.1107/S1600536809047084

**Published:** 2009-11-14

**Authors:** Primjira P. Tadbuppa, Edward R. T. Tiekink

**Affiliations:** a Department of Chemistry, National University of Singapore, Singapore 117543; b Department of Chemistry, University of Malaya, 50603 Kuala Lumpur, Malaysia

## Abstract

In the title compound, [Au(C_7_H_14_NOS)(C_18_H_33_P)], the Au^I^ atom is coordinated within an *S*,*P*-donor set that defines a slightly distorted linear geometry [S—Au—P = 174.94 (2)°], with the distortion due to a short intra­molecular Au⋯O contact [2.908 (2) Å].

## Related literature

For structural systematics and luminescence properties of phosphinegold(I) carbonimidothio­ates, see: Ho *et al.* (2006 ▶); Ho & Tiekink (2007 ▶); Kuan *et al.* (2008 ▶). For the synthesis, see: Hall *et al.* (1993 ▶).
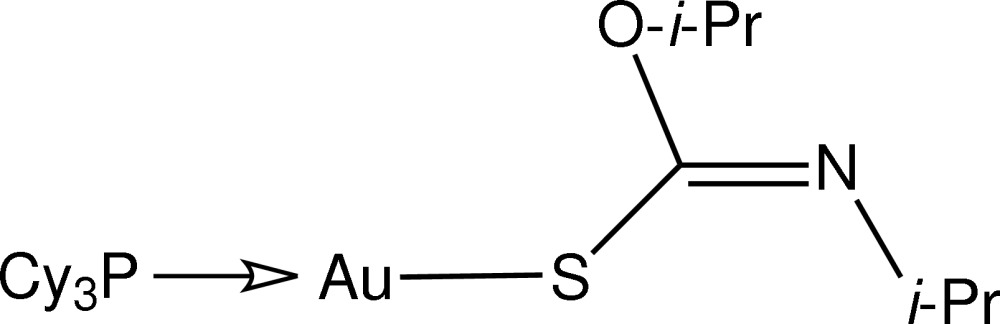


## Experimental

### 

#### Crystal data

[Au(C_7_H_14_NOS)(C_18_H_33_P)]
*M* *_r_* = 637.63
Triclinic, 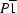

*a* = 11.1236 (5) Å
*b* = 11.7949 (6) Å
*c* = 11.9037 (6) Å
α = 73.869 (1)°
β = 85.282 (1)°
γ = 66.515 (1)°
*V* = 1375.30 (12) Å^3^
*Z* = 2
Mo *K*α radiation
μ = 5.50 mm^−1^
*T* = 223 K
0.19 × 0.16 × 0.11 mm

#### Data collection

Bruker SMART CCD diffractometer
Absorption correction: multi-scan (*SADABS*; Bruker, 2000 ▶) *T*_min_ = 0.636, *T*_max_ = 1
10973 measured reflections
6300 independent reflections
5853 reflections with *I* > 2σ(*I*)
*R*_int_ = 0.020

#### Refinement

*R*[*F* ^2^ > 2σ(*F* ^2^)] = 0.021
*wR*(*F* ^2^) = 0.054
*S* = 0.98
6300 reflections
271 parameters
H-atom parameters constrained
Δρ_max_ = 1.01 e Å^−3^
Δρ_min_ = −0.78 e Å^−3^

### 

Data collection: *SMART* (Bruker, 2000 ▶); cell refinement: *SAINT* (Bruker, 2000 ▶); data reduction: *SAINT*; program(s) used to solve structure: *PATTY* in *DIRDIF92* (Beurskens *et al.*, 1992 ▶); program(s) used to refine structure: *SHELXL97* (Sheldrick, 2008 ▶); molecular graphics: *DIAMOND* (Brandenburg, 2006 ▶); software used to prepare material for publication: *SHELXL97*.

## Supplementary Material

Crystal structure: contains datablocks global, I. DOI: 10.1107/S1600536809047084/hb5218sup1.cif

Structure factors: contains datablocks I. DOI: 10.1107/S1600536809047084/hb5218Isup2.hkl

Additional supplementary materials:  crystallographic information; 3D view; checkCIF report

## Figures and Tables

**Table 1:** Selected bond lengths (Å)

Au—S1	2.3091 (7)
Au—P1	2.2602 (7)

**Fig. 1.**
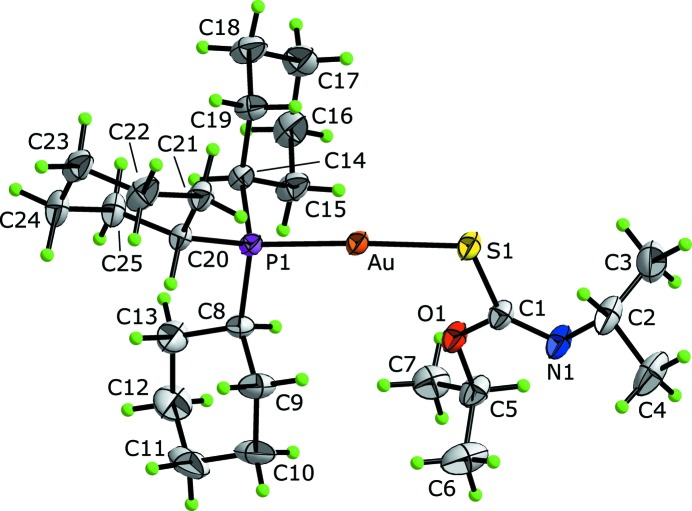
Molecular structure of (I) showing displacement ellipsoids at the 50% probability level.

## supplementary crystallographic information

### Comment

As a part of an on-going study of the structural systematics of molecules related to the general formula *R*_3_PAu[SC(OR')NR''] for *R*, *R*' and *R*'' = alkyl and aryl (Ho *et al.* 2006; Ho & Tiekink, 2007; Kuan *et al.*, 2008), the title compound, (I), was investigated.

In keeping with previous studies, the gold atom exists within an SP donor set defined by the phosphine-P and thiolate-S atoms, Table 1 and Fig. 1. Confirmation that the carbonimidothioate ligand is functioning as a thiolate is found in the magnitudes of the C1—S1 and C1═N1 distances of 1.767 (3) and 1.263 (3) Å, respectively. The coordination geometry is distorted from the ideal linear [S—Au—P = 174.94 (2) °] owing to the close approach of the O1 atom, 2.908 (2) Å.

### Experimental

Compound (I) was prepared following the standard literature procedure from the reaction of Cy_3_PAuCl and (^i^Pr)OC(S)N(H)(^i^Pr) in the presence of base (Hall *et al.*, 1993).

### Refinement

The H atoms were geometrically placed (C—H = 0.97–0.99 Å) and refined as riding with *U*_iso_(H) = 1.2–1.5*U*_eq_(C). The maximum and minimum residual electron density peaks of 1.01 and 0.78 e Å^-3^, respectively, were located 0.92 Å and 0.83 Å from the Au atom.

### Crystal data

**Table:**

[Au(C_7_H_14_NOS)(C_18_H_33_P)]	*Z* = 2
*M**_r_* = 637.63	*F*(000) = 644
Triclinic, *P*1	*D*_x_ = 1.540 Mg m^−^^3^
Hall symbol: -P 1	Mo *K*α radiation, λ = 0.71069 Å
*a* = 11.1236 (5) Å	Cell parameters from 7451 reflections
*b* = 11.7949 (6) Å	θ = 2.2–30.0°
*c* = 11.9037 (6) Å	µ = 5.50 mm^−^^1^
α = 73.869 (1)°	*T* = 223 K
β = 85.282 (1)°	Block, colourless
γ = 66.515 (1)°	0.19 × 0.16 × 0.11 mm
*V* = 1375.30 (12) Å^3^	

### Data collection

**Table:**

Bruker SMART CCD diffractometer	6300 independent reflections
Radiation source: fine-focus sealed tube	5853 reflections with *I* > 2σ(*I*)
graphite	*R*_int_ = 0.020
ω scans	θ_max_ = 27.5°, θ_min_ = 1.8°
Absorption correction: multi-scan (*SADABS*; Bruker, 2000)	*h* = −14→14
*T*_min_ = 0.636, *T*_max_ = 1	*k* = −15→15
10973 measured reflections	*l* = −13→15

### Refinement

**Table:**

Refinement on *F*^2^	Primary atom site location: structure-invariant direct methods
Least-squares matrix: full	Secondary atom site location: difference Fourier map
*R*[*F*^2^ > 2σ(*F*^2^)] = 0.021	Hydrogen site location: inferred from neighbouring sites
*wR*(*F*^2^) = 0.054	H-atom parameters constrained
*S* = 0.98	*w* = 1/[σ^2^(*F*_o_^2^) + (0.0323*P*)^2^] where *P* = (*F*_o_^2^ + 2*F*_c_^2^)/3
6300 reflections	(Δ/σ)_max_ = 0.001
271 parameters	Δρ_max_ = 1.01 e Å^−^^3^
0 restraints	Δρ_min_ = −0.78 e Å^−^^3^

### Special details

**Table:**

Geometry. All s.u.'s (except the s.u. in the dihedral angle between two l.s. planes) are estimated using the full covariance matrix. The cell s.u.'s are taken into account individually in the estimation of s.u.'s in distances, angles and torsion angles; correlations between s.u.'s in cell parameters are only used when they are defined by crystal symmetry. An approximate (isotropic) treatment of cell s.u.'s is used for estimating s.u.'s involving l.s. planes.
Refinement. Refinement of *F*^2^ against ALL reflections. The weighted *R*-factor *wR* and goodness of fit *S* are based on *F*^2^, conventional *R*-factors *R* are based on *F*, with *F* set to zero for negative *F*^2^. The threshold expression of *F*^2^ > 2σ(*F*^2^) is used only for calculating *R*-factors(gt) *etc*. and is not relevant to the choice of reflections for refinement. *R*-factors based on *F*^2^ are statistically about twice as large as those based on *F*, and *R*- factors based on ALL data will be even larger.

### Fractional atomic coordinates and isotropic or equivalent isotropic displacement parameters (Å^2^)

**Table:**

	*x*	*y*	*z*	*U*_iso_*/*U*_eq_	
Au	0.099579 (9)	0.244875 (8)	−0.000750 (8)	0.02575 (4)	
S1	0.17603 (7)	0.13648 (7)	0.18959 (6)	0.03236 (15)	
P1	0.04149 (6)	0.33925 (6)	−0.19218 (6)	0.02396 (13)	
O1	0.30086 (19)	−0.01420 (18)	0.05751 (16)	0.0341 (4)	
N1	0.3452 (2)	−0.1149 (2)	0.2521 (2)	0.0380 (6)	
C1	0.2845 (3)	−0.0148 (3)	0.1727 (2)	0.0302 (6)	
C2	0.3294 (3)	−0.1112 (3)	0.3740 (3)	0.0452 (8)	
H2	0.2426	−0.0446	0.3820	0.054*	
C3	0.4361 (4)	−0.0765 (4)	0.4098 (3)	0.0602 (10)	
H3A	0.4276	0.0076	0.3614	0.090*	
H3B	0.5216	−0.1397	0.3996	0.090*	
H3C	0.4270	−0.0753	0.4913	0.090*	
C4	0.3361 (4)	−0.2409 (4)	0.4495 (3)	0.0616 (11)	
H4A	0.2668	−0.2597	0.4247	0.092*	
H4B	0.3253	−0.2391	0.5307	0.092*	
H4C	0.4205	−0.3068	0.4412	0.092*	
C5	0.3930 (3)	−0.1300 (3)	0.0295 (3)	0.0385 (7)	
H5	0.4687	−0.1722	0.0859	0.046*	
C6	0.3260 (4)	−0.2196 (4)	0.0361 (4)	0.0618 (11)	
H6A	0.2994	−0.2453	0.1156	0.093*	
H6B	0.3860	−0.2950	0.0133	0.093*	
H6C	0.2494	−0.1766	−0.0163	0.093*	
C7	0.4384 (3)	−0.0863 (3)	−0.0916 (3)	0.0458 (7)	
H7A	0.4820	−0.0298	−0.0909	0.069*	
H7B	0.3634	−0.0407	−0.1459	0.069*	
H7C	0.4992	−0.1604	−0.1159	0.069*	
C8	0.0941 (3)	0.2106 (3)	−0.2671 (2)	0.0301 (5)	
H8	0.1905	0.1670	−0.2545	0.036*	
C9	0.0396 (4)	0.1087 (3)	−0.2087 (3)	0.0438 (7)	
H9A	−0.0562	0.1465	−0.2185	0.053*	
H9B	0.0619	0.0785	−0.1247	0.053*	
C10	0.0966 (4)	−0.0041 (3)	−0.2628 (4)	0.0544 (9)	
H10A	0.1909	−0.0483	−0.2437	0.065*	
H10B	0.0556	−0.0651	−0.2287	0.065*	
C11	0.0755 (4)	0.0378 (4)	−0.3945 (4)	0.0631 (11)	
H11A	0.1189	−0.0367	−0.4258	0.076*	
H11B	−0.0186	0.0722	−0.4136	0.076*	
C12	0.1290 (5)	0.1389 (4)	−0.4514 (3)	0.0652 (11)	
H12A	0.1084	0.1682	−0.5357	0.078*	
H12B	0.2246	0.1018	−0.4401	0.078*	
C13	0.0697 (4)	0.2527 (3)	−0.3988 (3)	0.0509 (8)	
H13A	0.1083	0.3154	−0.4344	0.061*	
H13B	−0.0249	0.2944	−0.4164	0.061*	
C14	0.1314 (2)	0.4419 (2)	−0.2571 (2)	0.0274 (5)	
H14	0.1062	0.4802	−0.3413	0.033*	
C15	0.2799 (3)	0.3628 (3)	−0.2449 (3)	0.0393 (7)	
H15A	0.3039	0.3165	−0.1626	0.047*	
H15B	0.3028	0.2992	−0.2897	0.047*	
C16	0.3585 (3)	0.4462 (3)	−0.2882 (3)	0.0469 (8)	
H16A	0.4522	0.3926	−0.2736	0.056*	
H16B	0.3432	0.4840	−0.3728	0.056*	
C17	0.3205 (3)	0.5522 (3)	−0.2277 (3)	0.0471 (8)	
H17A	0.3687	0.6069	−0.2609	0.057*	
H17B	0.3450	0.5144	−0.1443	0.057*	
C18	0.1739 (3)	0.6335 (3)	−0.2416 (3)	0.0449 (8)	
H18A	0.1508	0.6788	−0.3243	0.054*	
H18B	0.1515	0.6978	−0.1976	0.054*	
C19	0.0950 (3)	0.5502 (3)	−0.1972 (3)	0.0345 (6)	
H19A	0.0013	0.6042	−0.2114	0.041*	
H19B	0.1106	0.5129	−0.1126	0.041*	
C20	−0.1345 (2)	0.4342 (2)	−0.2282 (2)	0.0276 (5)	
H20	−0.1723	0.3730	−0.2346	0.033*	
C21	−0.2064 (3)	0.4929 (3)	−0.1287 (2)	0.0329 (6)	
H21A	−0.1926	0.4245	−0.0559	0.039*	
H21B	−0.1698	0.5519	−0.1167	0.039*	
C22	−0.3537 (3)	0.5655 (3)	−0.1575 (3)	0.0438 (7)	
H22A	−0.3920	0.5046	−0.1603	0.053*	
H22B	−0.3957	0.6063	−0.0953	0.053*	
C23	−0.3806 (3)	0.6671 (3)	−0.2735 (3)	0.0418 (7)	
H23A	−0.4754	0.7097	−0.2904	0.050*	
H23B	−0.3485	0.7320	−0.2690	0.050*	
C24	−0.3136 (3)	0.6074 (3)	−0.3709 (3)	0.0467 (8)	
H24A	−0.3305	0.6745	−0.4450	0.056*	
H24B	−0.3499	0.5466	−0.3787	0.056*	
C25	−0.1655 (3)	0.5375 (3)	−0.3458 (3)	0.0378 (7)	
H25A	−0.1283	0.5997	−0.3443	0.045*	
H25B	−0.1246	0.4974	−0.4086	0.045*	

### Atomic displacement parameters (Å^2^)

**Table:**

	*U*^11^	*U*^22^	*U*^33^	*U*^12^	*U*^13^	*U*^23^
Au	0.02761 (6)	0.01936 (6)	0.02462 (6)	−0.00442 (4)	−0.00234 (4)	−0.00356 (4)
S1	0.0360 (4)	0.0243 (3)	0.0259 (3)	−0.0015 (3)	−0.0027 (3)	−0.0044 (3)
P1	0.0252 (3)	0.0187 (3)	0.0247 (3)	−0.0053 (3)	−0.0015 (2)	−0.0049 (2)
O1	0.0358 (10)	0.0254 (10)	0.0282 (10)	−0.0006 (8)	0.0011 (8)	−0.0048 (8)
N1	0.0385 (13)	0.0281 (13)	0.0303 (13)	0.0008 (11)	0.0010 (10)	−0.0025 (10)
C1	0.0285 (13)	0.0268 (14)	0.0289 (14)	−0.0059 (11)	0.0021 (10)	−0.0054 (11)
C2	0.0455 (18)	0.0347 (17)	0.0309 (16)	0.0037 (14)	0.0022 (13)	−0.0008 (13)
C3	0.064 (2)	0.058 (2)	0.044 (2)	−0.011 (2)	−0.0136 (17)	−0.0071 (17)
C4	0.064 (2)	0.044 (2)	0.046 (2)	−0.0061 (18)	0.0062 (17)	0.0104 (16)
C5	0.0369 (15)	0.0297 (15)	0.0375 (16)	−0.0022 (13)	0.0065 (12)	−0.0097 (12)
C6	0.084 (3)	0.041 (2)	0.062 (3)	−0.029 (2)	0.030 (2)	−0.0193 (18)
C7	0.0444 (18)	0.0478 (19)	0.0451 (19)	−0.0154 (16)	0.0150 (14)	−0.0201 (15)
C8	0.0338 (14)	0.0249 (13)	0.0318 (14)	−0.0083 (11)	0.0003 (11)	−0.0126 (11)
C9	0.058 (2)	0.0347 (17)	0.0484 (19)	−0.0249 (16)	0.0116 (15)	−0.0194 (14)
C10	0.061 (2)	0.0403 (19)	0.078 (3)	−0.0250 (18)	0.0040 (19)	−0.0323 (19)
C11	0.059 (2)	0.062 (2)	0.080 (3)	−0.012 (2)	−0.006 (2)	−0.052 (2)
C12	0.094 (3)	0.058 (2)	0.042 (2)	−0.018 (2)	0.0045 (19)	−0.0297 (18)
C13	0.072 (2)	0.0406 (18)	0.0328 (17)	−0.0105 (17)	−0.0007 (15)	−0.0158 (14)
C14	0.0288 (13)	0.0221 (12)	0.0286 (13)	−0.0088 (11)	−0.0002 (10)	−0.0043 (10)
C15	0.0294 (14)	0.0299 (15)	0.0532 (19)	−0.0065 (12)	0.0039 (12)	−0.0112 (13)
C16	0.0291 (15)	0.0450 (19)	0.061 (2)	−0.0141 (14)	0.0047 (14)	−0.0079 (16)
C17	0.0380 (17)	0.0453 (19)	0.062 (2)	−0.0241 (16)	0.0003 (15)	−0.0093 (16)
C18	0.0431 (17)	0.0322 (16)	0.063 (2)	−0.0198 (15)	0.0036 (15)	−0.0102 (15)
C19	0.0337 (14)	0.0280 (14)	0.0434 (17)	−0.0125 (12)	0.0055 (12)	−0.0127 (12)
C20	0.0252 (12)	0.0233 (13)	0.0297 (14)	−0.0053 (10)	−0.0047 (10)	−0.0046 (10)
C21	0.0297 (14)	0.0304 (14)	0.0325 (15)	−0.0060 (12)	0.0000 (11)	−0.0075 (11)
C22	0.0282 (15)	0.0349 (17)	0.057 (2)	−0.0050 (13)	0.0057 (13)	−0.0063 (14)
C23	0.0271 (14)	0.0313 (16)	0.056 (2)	−0.0050 (12)	−0.0045 (13)	−0.0038 (14)
C24	0.0392 (17)	0.0387 (18)	0.0473 (19)	−0.0044 (14)	−0.0180 (14)	0.0003 (14)
C25	0.0351 (15)	0.0366 (16)	0.0298 (15)	−0.0056 (13)	−0.0067 (12)	−0.0012 (12)

### Geometric parameters (Å, °)

**Table:**

Au—S1	2.3091 (7)	C12—C13	1.524 (5)
Au—P1	2.2602 (7)	C12—H12A	0.9800
S1—C1	1.767 (3)	C12—H12B	0.9800
P1—C20	1.842 (3)	C13—H13A	0.9800
P1—C14	1.839 (2)	C13—H13B	0.9800
P1—C8	1.844 (3)	C14—C19	1.532 (4)
O1—C1	1.367 (3)	C14—C15	1.535 (4)
O1—C5	1.452 (3)	C14—H14	0.9900
N1—C1	1.263 (3)	C15—C16	1.525 (4)
N1—C2	1.458 (4)	C15—H15A	0.9800
C2—C4	1.520 (4)	C15—H15B	0.9800
C2—C3	1.524 (5)	C16—C17	1.513 (5)
C2—H2	0.9900	C16—H16A	0.9800
C3—H3A	0.9700	C16—H16B	0.9800
C3—H3B	0.9700	C17—C18	1.522 (4)
C3—H3C	0.9700	C17—H17A	0.9800
C4—H4A	0.9700	C17—H17B	0.9800
C4—H4B	0.9700	C18—C19	1.529 (4)
C4—H4C	0.9700	C18—H18A	0.9800
C5—C7	1.508 (4)	C18—H18B	0.9800
C5—C6	1.500 (5)	C19—H19A	0.9800
C5—H5	0.9900	C19—H19B	0.9800
C6—H6A	0.9700	C20—C25	1.536 (4)
C6—H6B	0.9700	C20—C21	1.543 (4)
C6—H6C	0.9700	C20—H20	0.9900
C7—H7A	0.9700	C21—C22	1.531 (4)
C7—H7B	0.9700	C21—H21A	0.9800
C7—H7C	0.9700	C21—H21B	0.9800
C8—C13	1.519 (4)	C22—C23	1.515 (4)
C8—C9	1.530 (4)	C22—H22A	0.9800
C8—H8	0.9900	C22—H22B	0.9800
C9—C10	1.525 (5)	C23—C24	1.512 (5)
C9—H9A	0.9800	C23—H23A	0.9800
C9—H9B	0.9800	C23—H23B	0.9800
C10—C11	1.514 (6)	C24—C25	1.532 (4)
C10—H10A	0.9800	C24—H24A	0.9800
C10—H10B	0.9800	C24—H24B	0.9800
C11—C12	1.512 (6)	C25—H25A	0.9800
C11—H11A	0.9800	C25—H25B	0.9800
C11—H11B	0.9800		
			
P1—Au—S1	174.94 (2)	C8—C13—C12	111.3 (3)
C1—S1—Au	101.17 (9)	C8—C13—H13A	109.4
C20—P1—C14	108.31 (12)	C12—C13—H13A	109.4
C20—P1—C8	106.97 (12)	C8—C13—H13B	109.4
C14—P1—C8	106.64 (12)	C12—C13—H13B	109.4
C20—P1—Au	116.82 (9)	H13A—C13—H13B	108.0
C14—P1—Au	110.30 (9)	C19—C14—C15	110.1 (2)
C8—P1—Au	107.28 (9)	C19—C14—P1	109.76 (18)
C1—O1—C5	118.2 (2)	C15—C14—P1	110.57 (18)
C1—N1—C2	119.2 (3)	C19—C14—H14	108.8
N1—C1—O1	120.5 (3)	C15—C14—H14	108.8
N1—C1—S1	127.7 (2)	P1—C14—H14	108.8
O1—C1—S1	111.82 (18)	C16—C15—C14	112.3 (2)
N1—C2—C4	109.0 (3)	C16—C15—H15A	109.1
N1—C2—C3	109.0 (3)	C14—C15—H15A	109.1
C4—C2—C3	111.7 (3)	C16—C15—H15B	109.1
N1—C2—H2	109.0	C14—C15—H15B	109.1
C4—C2—H2	109.0	H15A—C15—H15B	107.9
C3—C2—H2	109.0	C17—C16—C15	111.5 (3)
C2—C3—H3A	109.5	C17—C16—H16A	109.3
C2—C3—H3B	109.5	C15—C16—H16A	109.3
H3A—C3—H3B	109.5	C17—C16—H16B	109.3
C2—C3—H3C	109.5	C15—C16—H16B	109.3
H3A—C3—H3C	109.5	H16A—C16—H16B	108.0
H3B—C3—H3C	109.5	C16—C17—C18	111.4 (3)
C2—C4—H4A	109.5	C16—C17—H17A	109.3
C2—C4—H4B	109.5	C18—C17—H17A	109.3
H4A—C4—H4B	109.5	C16—C17—H17B	109.3
C2—C4—H4C	109.5	C18—C17—H17B	109.3
H4A—C4—H4C	109.5	H17A—C17—H17B	108.0
H4B—C4—H4C	109.5	C17—C18—C19	110.9 (3)
O1—C5—C7	105.7 (2)	C17—C18—H18A	109.5
O1—C5—C6	109.4 (3)	C19—C18—H18A	109.5
C7—C5—C6	112.7 (3)	C17—C18—H18B	109.5
O1—C5—H5	109.6	C19—C18—H18B	109.5
C7—C5—H5	109.6	H18A—C18—H18B	108.0
C6—C5—H5	109.6	C18—C19—C14	112.2 (2)
C5—C6—H6A	109.5	C18—C19—H19A	109.2
C5—C6—H6B	109.5	C14—C19—H19A	109.2
H6A—C6—H6B	109.5	C18—C19—H19B	109.2
C5—C6—H6C	109.5	C14—C19—H19B	109.2
H6A—C6—H6C	109.5	H19A—C19—H19B	107.9
H6B—C6—H6C	109.5	C25—C20—C21	110.5 (2)
C5—C7—H7A	109.5	C25—C20—P1	114.90 (18)
C5—C7—H7B	109.5	C21—C20—P1	111.93 (17)
H7A—C7—H7B	109.5	C25—C20—H20	106.3
C5—C7—H7C	109.5	C21—C20—H20	106.3
H7A—C7—H7C	109.5	P1—C20—H20	106.3
H7B—C7—H7C	109.5	C22—C21—C20	111.2 (2)
C13—C8—C9	111.0 (3)	C22—C21—H21A	109.4
C13—C8—P1	116.5 (2)	C20—C21—H21A	109.4
C9—C8—P1	111.1 (2)	C22—C21—H21B	109.4
C13—C8—H8	105.8	C20—C21—H21B	109.4
C9—C8—H8	105.8	H21A—C21—H21B	108.0
P1—C8—H8	105.8	C23—C22—C21	111.7 (2)
C8—C9—C10	110.3 (3)	C23—C22—H22A	109.3
C8—C9—H9A	109.6	C21—C22—H22A	109.3
C10—C9—H9A	109.6	C23—C22—H22B	109.3
C8—C9—H9B	109.6	C21—C22—H22B	109.3
C10—C9—H9B	109.6	H22A—C22—H22B	107.9
H9A—C9—H9B	108.1	C24—C23—C22	110.5 (3)
C11—C10—C9	112.2 (3)	C24—C23—H23A	109.5
C11—C10—H10A	109.2	C22—C23—H23A	109.5
C9—C10—H10A	109.2	C24—C23—H23B	109.5
C11—C10—H10B	109.2	C22—C23—H23B	109.5
C9—C10—H10B	109.2	H23A—C23—H23B	108.1
H10A—C10—H10B	107.9	C23—C24—C25	110.8 (3)
C12—C11—C10	111.6 (3)	C23—C24—H24A	109.5
C12—C11—H11A	109.3	C25—C24—H24A	109.5
C10—C11—H11A	109.3	C23—C24—H24B	109.5
C12—C11—H11B	109.3	C25—C24—H24B	109.5
C10—C11—H11B	109.3	H24A—C24—H24B	108.1
H11A—C11—H11B	108.0	C24—C25—C20	111.3 (2)
C11—C12—C13	110.6 (3)	C24—C25—H25A	109.4
C11—C12—H12A	109.5	C20—C25—H25A	109.4
C13—C12—H12A	109.5	C24—C25—H25B	109.4
C11—C12—H12B	109.5	C20—C25—H25B	109.4
C13—C12—H12B	109.5	H25A—C25—H25B	108.0
H12A—C12—H12B	108.1		
			
C2—N1—C1—O1	177.8 (2)	Au—P1—C14—C19	−62.62 (19)
C2—N1—C1—S1	−0.8 (4)	C20—P1—C14—C15	−172.0 (2)
C5—O1—C1—N1	−2.1 (4)	C8—P1—C14—C15	−57.1 (2)
C5—O1—C1—S1	176.71 (19)	Au—P1—C14—C15	59.0 (2)
Au—S1—C1—N1	−173.7 (2)	C19—C14—C15—C16	−53.6 (3)
Au—S1—C1—O1	7.64 (19)	P1—C14—C15—C16	−175.1 (2)
C1—N1—C2—C4	146.0 (3)	C14—C15—C16—C17	55.0 (4)
C1—N1—C2—C3	−91.8 (3)	C15—C16—C17—C18	−55.6 (4)
C1—O1—C5—C7	−153.8 (2)	C16—C17—C18—C19	55.7 (4)
C1—O1—C5—C6	84.5 (3)	C17—C18—C19—C14	−55.6 (4)
C20—P1—C8—C13	56.3 (3)	C15—C14—C19—C18	54.1 (3)
C14—P1—C8—C13	−59.4 (3)	P1—C14—C19—C18	176.0 (2)
Au—P1—C8—C13	−177.6 (2)	C14—P1—C20—C25	31.2 (2)
C20—P1—C8—C9	−72.0 (2)	C8—P1—C20—C25	−83.4 (2)
C14—P1—C8—C9	172.2 (2)	Au—P1—C20—C25	156.48 (18)
Au—P1—C8—C9	54.1 (2)	C14—P1—C20—C21	−95.9 (2)
C13—C8—C9—C10	55.2 (4)	C8—P1—C20—C21	149.53 (18)
P1—C8—C9—C10	−173.5 (2)	Au—P1—C20—C21	29.4 (2)
C8—C9—C10—C11	−54.5 (4)	C25—C20—C21—C22	53.5 (3)
C9—C10—C11—C12	55.0 (4)	P1—C20—C21—C22	−177.03 (19)
C10—C11—C12—C13	−55.3 (5)	C20—C21—C22—C23	−55.3 (3)
C9—C8—C13—C12	−57.0 (4)	C21—C22—C23—C24	57.4 (4)
P1—C8—C13—C12	174.6 (3)	C22—C23—C24—C25	−58.0 (3)
C11—C12—C13—C8	56.6 (4)	C23—C24—C25—C20	57.3 (4)
C20—P1—C14—C19	66.4 (2)	C21—C20—C25—C24	−54.7 (3)
C8—P1—C14—C19	−178.80 (19)	P1—C20—C25—C24	177.5 (2)
